# Weekly, seasonal and holiday body weight fluctuation patterns among individuals engaged in a European multi-centre behavioural weight loss maintenance intervention

**DOI:** 10.1371/journal.pone.0232152

**Published:** 2020-04-30

**Authors:** Jake Turicchi, Ruairi O’Driscoll, Graham Horgan, Cristiana Duarte, Antonio L. Palmeira, Sofus C. Larsen, Berit L. Heitmann, James Stubbs

**Affiliations:** 1 Appetite Control and Energy Balance Group, School of Psychology, University of Leeds, Leeds, United Kingdom; 2 Biomathematics & Statistics Scotland, Aberdeen, United Kingdom; 3 Faculdade de Motricidade Humana, Universidade de Lisboa, Lisbon, Portugal; 4 Research Unit for Dietary Studies, The Parker Institute, Bispebjerg and Frederiksberg Hospital, The Capital Region, Copenhagen, Denmark; 5 The Boden Institute of Obesity, Nutrition and Eating disorder, University of Sydney, Sydney, Australia; 6 Department of Public Health, Section for General Medicine, University of Copenhagen, Copenhagen, Denmark; Universidad de Tarapaca, CHILE

## Abstract

**Background:**

Technological advances in remote monitoring offer new opportunities to quantify body weight patterns in free-living populations. This paper describes body weight fluctuation patterns in response to weekly, holiday (Christmas) and seasonal time periods in a large group of individuals engaged in a weight loss maintenance intervention.

**Methods:**

Data was collected as part The NoHoW Project which was a pan-European weight loss maintenance trial. Three eligible groups were defined for weekly, holiday and seasonal analyses, resulting in inclusion of 1,421, 1,062 and 1,242 participants, respectively. Relative weight patterns were modelled on a time series following removal of trends and grouped by gender, country, BMI and age.

**Results:**

Within-week fluctuations of 0.35% were observed, characterised by weekend weight gain and weekday reduction which differed between all groups. Over the Christmas period, weight increased by a mean 1.35% and was not fully compensated for in following months, with some differences between countries observed. Seasonal patterns were primarily characterised by the effect of Christmas weight gain and generally not different between groups.

**Conclusions:**

This evidence may improve current understanding of regular body weight fluctuation patterns and help target future weight management interventions towards periods, and in groups, where weight gain is anticipated.

## Introduction

Weight gain occur across the adult lifespan, at reported rates of around 0.2–1 kg/year [1,2] in a non-linear manner [3] which, in addition to the physiological effects of ageing (e.g. reduced resting metabolic rate [4] and adipose lipid turnover [5]), may be a response to short periods of increased energy intake (EI) and/or decreased energy expenditure (EE) which are not subsequently compensated for [6]. These periods are often influenced by temporal factors (e.g. weekends or holidays) that cue changes in energy balance behaviours in the short term and may consequently produce longer-term weight gain [7–9] if not subsequently compensated for. Two features of body weight, the trend (i.e. the longer-term weight change component) and the variability (i.e. the shorter-term fluctuation component) can be identified but are not necessarily related. While the trend in body weight can be estimated with relative accuracy using infrequent measurements of body weight, less is understood about regular body weight variability in free-living adults due to difficulties in collecting frequent and valid measurements.

Temporal cues such as weekends, seasons and holidays are consistent and repeating therefore associations with body weight responses have been documented in a small number of studies. Within-week patterns have been reported previously, characterized by greater body weight at the weekend which declines during the week, with consistent patterns evident in Europe [10,11] and North America [12], a pattern which has been associated with longer-term weight gain [11]. Similarly, holiday (typically Christmas) weight gain has been well documented with increases in weight in the region of 0.4-1kg being reported consistently across the UK, Europe, North America and Japan [13]. Seasonal fluctuations across the year are often less pronounced and more inconsistent, though may be characterized by a decrease in weight during the summer and an increase in the winter [14–16] which has been shown in both Northern and Southern hemispheres [15]. Indeed, it is possible that in each of these cases, short-term increases in weight partially contribute to long-term weight gain. Currently, longitudinal studies with sufficient frequency and duration of weight data to fully investigate this effect are lacking.

Coinciding with weight changes, repeating patterns of energy balance behaviours have been described. Dietary energy density tends to increase (e.g. increased frequency of processed and fast food intake consumption) at weekends in many individuals [17–19], often accompanied by increased alcohol intake [20] though substantial individual variability exists. Weekly patterns of physical activity are less clear with some reports showing increased [21,22] or decreased [23,24] activity levels at weekends. Over the Christmas period, increases in energy and fat intake have been reported [25,26] and are likely accompanied by decreases in physical activity and increases in sedentary behavior [27]. Reports on seasonal patterns in energy balance behaviours are sparse, though increases in EI and decreases in physical activity have been reported in winter and autumn months [28,29]. Behavioural (and thus body weight) responses to the temporal environment may be dependent on individual differences in age, gender, BMI or country, though these effects have not been explored extensively using frequently measured body weight data.

Advances in technology have allowed for remote tracking of body weight in clinical and research environments. Where previously, identification of body weight patterns relied on infrequent or self-reported measurements, implementation of wifi-connected smart scales connected to online personal accounts has introduced new possibilities for body weight pattern recognition in clinical and research environments. Specifically, increased frequency of valid [30] and time-stamped measurements, reduction in potential biases in data collection (associated with self-report) and reduced participant burden accompany smart scale data collection. Furthermore, advanced data analysis protocols for processing and analysing the dense and complex data produced by these devices are becoming more accessible [10]. Such techniques include isolation of body weight variability patterns from linear and non-linear trends which may confound the specific outcomes of interest [11], such as risk of disease [31,32]. A recent pan-European multi-centre weight loss maintenance intervention (the NoHoW trial [33]) has collected body weight data over 18 months from a large sample of individuals provided with smart scales. The aim of the present paper is to improve the understanding of body weight variability by describing weekly, holiday and seasonal patterns according to gender, country, BMI and age groups using improved methods of data collection and data analysis.

## Methods

### Study design

The NoHoW trial is a 2x2 factorial randomised controlled trial testing the efficacy of a digital toolkit for promoting evidence-based behaviour change for weight loss maintenance structured around two conditions: (1) self-regulation and motivation and (2) contextual behavioural emotion regulation. It was delivered in three centres located in the United Kingdom (Leeds), Denmark (Copenhagen), and Portugal (Lisbon). A detailed description of the trial can be found elsewhere [33]. In short, eligible participants were randomised into 4 arms upon entry to the trial ((1) active control, (2) self-regulation and motivation, (3) contextual behavioural emotion regulation and (4) self-regulation, motivation and emotion regulation (i.e. arms 2 and 3 combined)). Participants from all arms were pooled for this analysis. All participants were provided with a Fitbit Aria body weight smart scale (Fitbit Inc, San Francisco, CA, USA) and a Fitbit Charge 2 activity monitor device (Fitbit Inc, San Francisco, CA, USA). Participants were instructed to weigh themselves at least twice per week and wear the activity monitor at all times apart from when charging and during activities involving water. No specific dietary or physical activity advice was given, though intervention content (which is provided in more detail elsewhere [33]) provided guidance on self-regulation and planning which may support a healthy lifestyle. Outcome measures were made at 6, 12 and 18 months. The trial is registered with the ISRCTN registry (ISRCTN88405328). The NoHoW study has received funding from the European Union's Horizon 2020 research and innovation programme (grant agreement number: 643309). Ethical approval has been granted by local institutional ethics committees at the Universities of Leeds (17–0082; 27-Feb-2017), Lisbon (17/2016; 20-Feb-2017) and the Capital Region of Denmark (H-16030495; 8-Mar-2017).

### Participants

**Fig 1:**
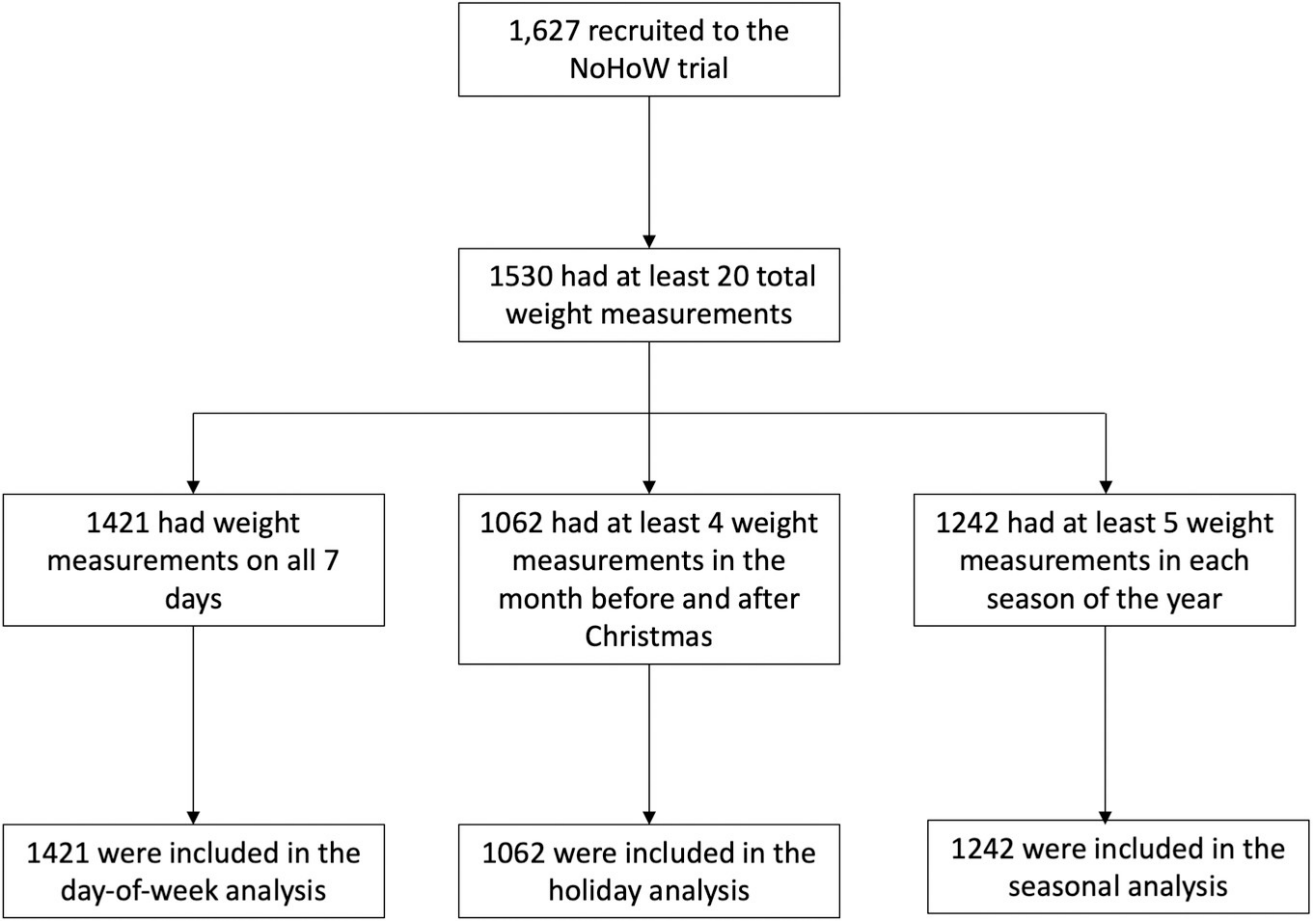
Participant flow diagram. Participant flow diagram illustrating inclusion of participants into each of the 3 analyses.

Inclusion and exclusion criteria for the NoHoW trial can be found in full elsewhere [33]. Briefly, individuals were eligible if they were aged 18 years or older, had verification of ≥5% weight loss in the 12 months prior to recruitment (excluding surgical weight loss) and had a BMI of ≥25 kg/m2 prior to weight loss. Participants were recruited between March 2017 and March 2018. Firstly, for inclusion in all present analyses, participants must have provided at least 20 weight measurements in one year. Additionally, for inclusion in the weekly analysis, at least one weight reading was required on each day of the week. For inclusion in the seasonal analysis, at least 5 weights were required in each season of the year. Seasons were defined as follows: Spring (20th March– 20th June); Summer (21st June– 22nd September), Autumn (23rd September– 20th December) and Winter (21st December– 19th March) based on astronomical dates for solstice and equinox occurrence in year 2019. For inclusion in the Christmas analysis, at least 4 weights were required in the 30 days prior to and after Christmas (defined as the 25th of December). These minimum criteria were designed to improve the accuracy of statistical smoothing as suggested previously [15] and also demonstrated previously by our group [34]. Inclusion in one sample did not prevent inclusion in another. A participant flow diagram is provided in Fig 1.

### Anthropometric measures

On the initial visit, body weight and height were measured using the SECA 704s combined stadiometer and electronic scale in a fasted state, first thing in the morning, in light clothing. From this, BMI was calculated [BMI = (body weight (kg))⁄(height (m)^2].

### Fitbit Aria scale

All participants were provided with a commercially available Fitbit Aria scale and advised to weigh themselves at least 2 times per week, first thing in the morning after emptying their bladder and with no or light clothing. The device shows excellent agreement with a calibrated research grade SECA 704s scale [30]. Data collected from the device was synchronised to a personal Fitbit account which participants could access on their phone, tablet or computer and data from each personal account was continuously streamed to the NoHoW data hub. From this, information on both the frequency of self-weighing and the absolute weight was collated for each individual. In the event of 2 or more weight measures in one day, we utilized the first recorded weight. Data was collected from the scales for up to 2 years.

### Statistical analysis

Three sub-samples were generated based on meeting eligibility criteria for each analysis (i.e. for weekly, Christmas and seasonal analyses). Participant characteristics are given as mean (standard error) or relative percentages (where specified) in Table 1 and scale use is described as completeness of data per day of the week and month of the year relative the amount of data possible for the given day or month. Change in scale use per week over 2 years was illustrated as mean (standard error) number of weights per week for each week in all participants from the entire sample. Body weight data was initially screened for outliers based on physiological plausibility of weight change (S1 Table) informed by rapid weight change which occurs under conditions of very low-calorie diets [35,36] or substantial overfeeding [37,38].

**Table 1:** Participant characteristics.

	Weekly analysis (n = 1,421)	Holiday analysis (n = 1,062)	Seasonal analysis (n = 1,242)
Gender = women (%)	982 (69.1)	749 (70.5)	865 (69.6)
Age group (%)			
under 30 years	164 (11.5)	100 (9.4)	132 (10.6)
30 to 45 years	618 (43.5)	440 (41.4)	530 (42.7)
46 to 60 years	506 (35.6)	404 (38.0)	451 (36.3)
over 60 years	133 (9.4)	118 (11.1)	129 (10.4)
Country (%)			
Denmark	474 (33.4)	386 (36.3)	412 (33.2)
Portugal	471 (33.1)	318 (29.9)	391 (31.5)
UK	476 (33.5)	358 (33.7)	439 (35.3)
BMI status (%)			
Healthy weight	263 (18.5)	195 (18.4)	224 (18.0)
Overweight	616 (43.3)	456 (42.9)	545 (43.9)
Obese C1	335 (23.6)	259 (24.4)	303 (24.4)
Obese C2-3	207 (14.6)	152 (14.3)	170 (13.7)
Weight (kg)	84.4 (0.4)	84.2 (0.5)	84.1 (0.5)
Duration (days)	566 (4.1)	603 (3.6)	607 (2.9)
Total weight measurements	220 (4.1)	262 (4.7)	243 (4.3)

Participant characteristics grouped by analysis (weekly, holiday and seasonal). Data provided as absolute means (SD) and relative percentage (within a given analysis) or as mean and standard error.

In all analyses, each individual’s body weight data was converted to a time-series and decomposed to remove the trend element (i.e. detrended). This process refers specifically to the process of removing the overall trajectory of the time series thus centering the body weight and leaving the variability. Detrending of body weight data was conducted to account for the potentially confounding effect of weight change on patterns of variability as suggested previously [11,15]. For weekly and Christmas analyses, the body weight data was detrended by fitting a LOESS (locally estimated scatterplot smoothing) regression (S2A Fig) to each participant. LOESS regression is a locally weighted, non-linear and non-parametric tool which can be used to employ quadratic polynomial models on a moving collection of data points (termed a “neighborhood”). LOESS regression was chosen to account for the non-linearity of weight change [39], allowing recognition of weekly and Christmas patterns independent of the trend (S2B Fig). The assigned ‘span’ value determines the size of the neighbourhood by which the LOESS regression is fit, whereby greater spans generate a smoother (or more linear) fit to the data, whereas lower spans generate a tighter fit to the data and may risk overfitting. The span was set at 0.5 for weekly analysis and 0.7 for the Christmas analysis based on visual inspection.

To identify seasonal patterns, a linear trend was fitted for the entire period measured for each participant. This was deemed optimal when examining variability over a long period (up to 2 years) as non-linear trends such as a LOESS regression are likely to capture the variability patterns of interest and therefore reduce seasonal fluctuations, whereas linear trends allow greater deviation from the trend (illustrated in S2 Fig). Next, the trends were subtracted from the observed weight. Following detrending, the detrended weights were converted to relative detrended weights which reflect the relative difference in weight between a given point and the trend, as done previously [15]. Within, we use the term “weight” to refer to this relative deviation from the trend.

To identify weekly patterns, we averaged the relative detrended weights for each day of the week, providing a value representing the mean relative deviation between the weight and the trend on each day. To identify seasonal and Christmas patterns, we imputed missing data using an exponentially weighted moving average (EWMA) from the TS Impute package [40] which used a moving window of 3 days each side of the central missing value (i.e. a 1-week EWMA). Imputation by EWMA was chosen based on a previous unpublished analysis by this group showing that it provided one of the best performances compared to true body weight data as compared by root mean square error (RMSE) and mean absolute percentage error (MAPE). Imputation was conducted for Christmas and seasonal analyses but not the within-week analysis because the smoothing effect of the moving average imputation reduces the differences between sequential days and therefore removes some of the variability, but this is not a concern when examining patterns over longer periods such as several months or years. This has been illustrated in S1 Fig. Lastly, for seasonal and Christmas analyses, we combined multiple years on to a year-less time axis and averaged each day of the year for all participants in each analysis.

For each analysis, we grouped individuals by gender, region, BMI and age groups to test for differences in variability patterns between baseline characteristics. All tests were conducted following data processing (e.g. detrending in addition to imputation for holiday and seasonal analyses). For the weekly analysis, we compared differences in each grouping variable for each day of the week. For the Christmas analysis, we calculated weight gain by taking the day where weight was lowest in the 1 month prior to Christmas and highest in the 1 month after Christmas and calculating the difference to define relative weight change (after detrending) in response to the holiday period. We then tested the difference in Christmas weight change by each grouping variable. For the seasonal analysis we grouped data by year, season and group then compared the difference in mean relative deviation for each season between groups. All group comparisons were made using a multi-factor one-way analysis of variance (ANOVA) with type III sum of squares adjusted for each grouping variable (gender, country, BMI status and age group). This method was chosen to deal with potentially unbalanced groups and for covariance between the independent variables. Next, we applied Tukey’s post-hoc to significant models to investigate specific differences between groups. Full multivariate ANOVA results can be found in S2–S4 Tables. All analyses were conducted in R version 3.5.1 (www.r-project.org).

## Results

**Fig 2:**
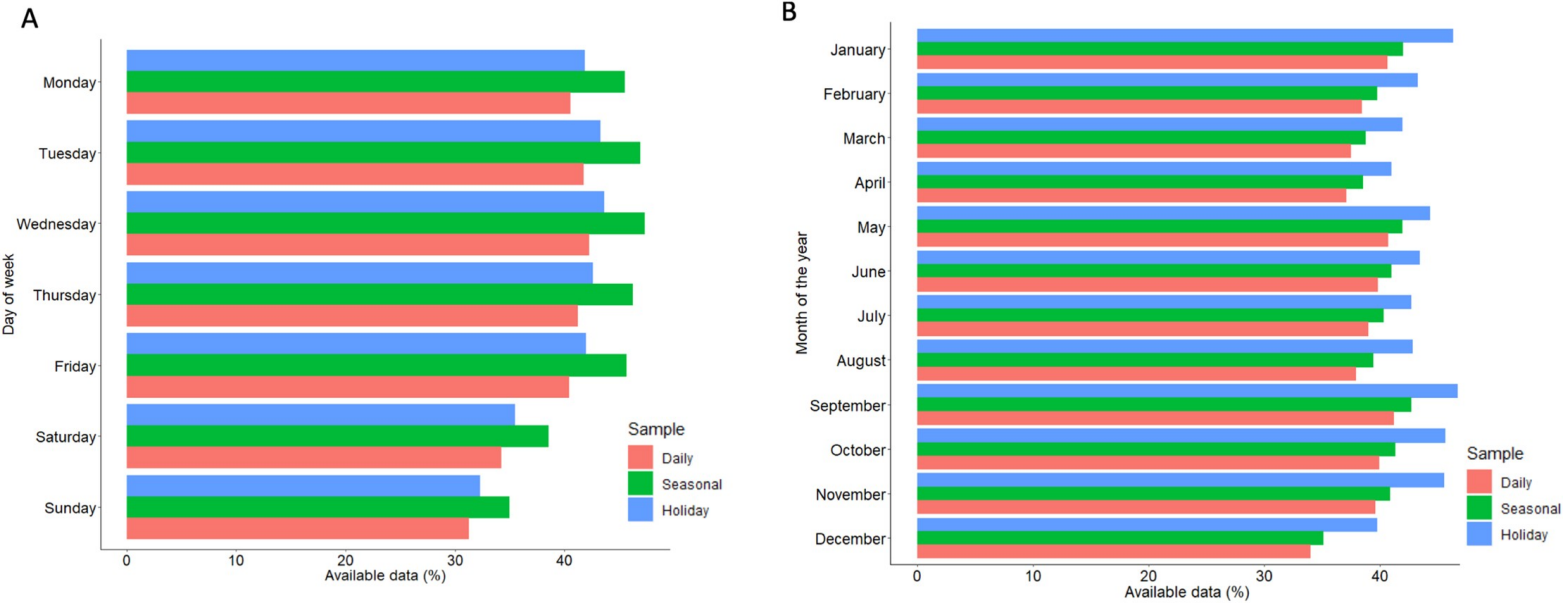
Frequency of scale use by day of week and month of year. Frequency of weight data collected, given for each analysis (daily, seasonal and holiday). Fig (A) shows completeness of data per day of the week relative to the total amount of data possible for the given day and fig (B) shows completeness of data per month of the year relative the amount of data possible for the given year.

**Fig 3:**
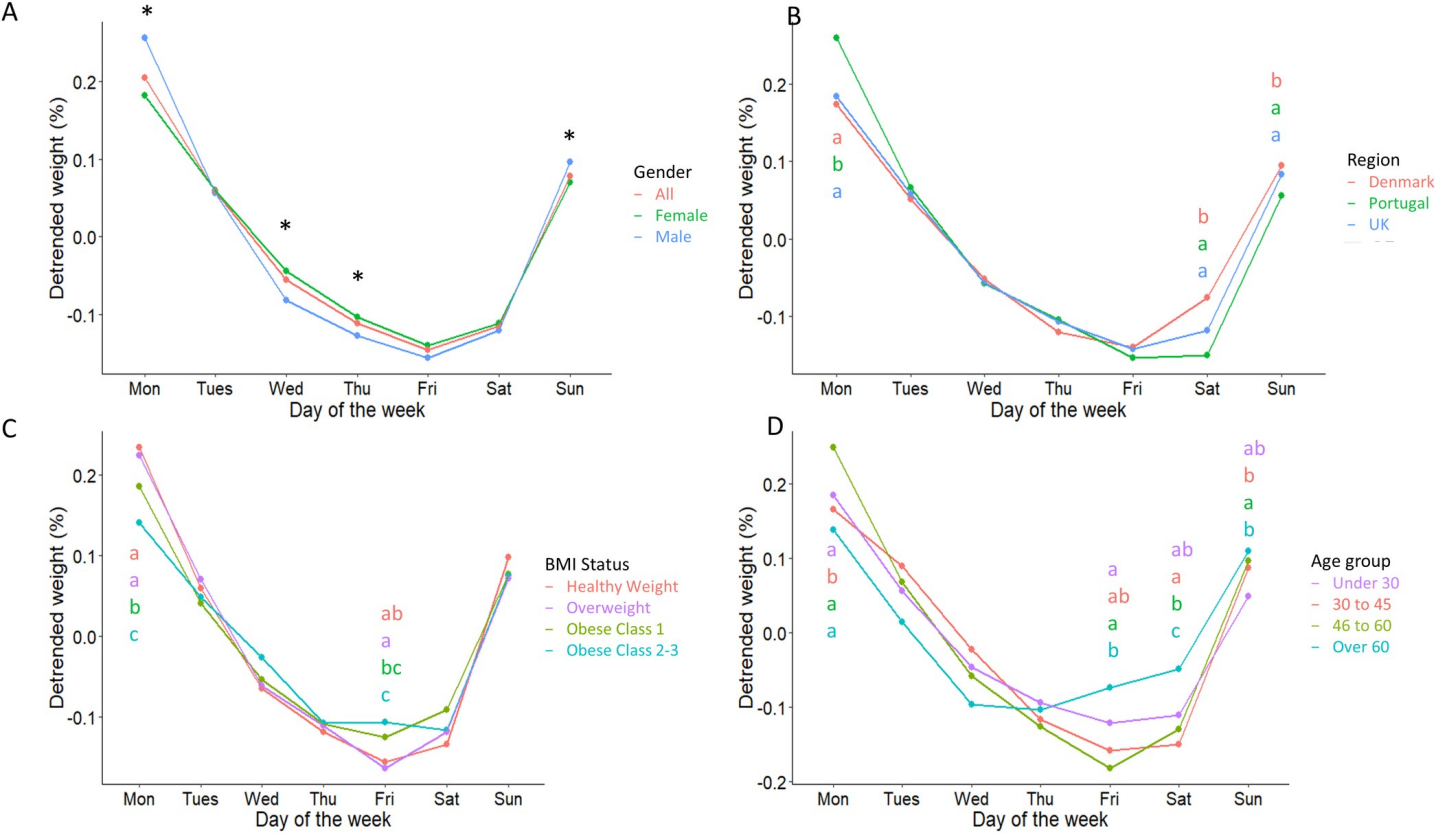
Scale use over the duration of the trial. Mean (standard error) scale use per week over 2 years for each week in all participants from the entire sample.

Participant characteristics for each analysis are given in Table 1. The weekly, Christmas and seasonal analyses included 1,421, 1,062 and 1,242 participants respectively. Participants in the weekly analysis weighed themselves on average 220 times over 566 days; in the Christmas analysis on average 262 times over 603 days and in the seasonal analysis on average 243 times over 607 days. Distribution of weight is given by day of the week (Fig 2A) and month of the year (Fig 2B) relative to total possible days. The greatest proportion of data was available on Tuesday and Wednesday, with the least available on Sunday and Saturday respectively. Per month, data was most complete in January and September to November, whereas December, April and March had the greatest proportion of missing data respectively. Self-weighing was averaged in relation to week of the trial for each participant (Fig 3), showing initial scale use of around 4 times per week which reduced to around 2.5 times per week over the course of the trial.

### Weekly patterns of body weight

**Fig 4:**
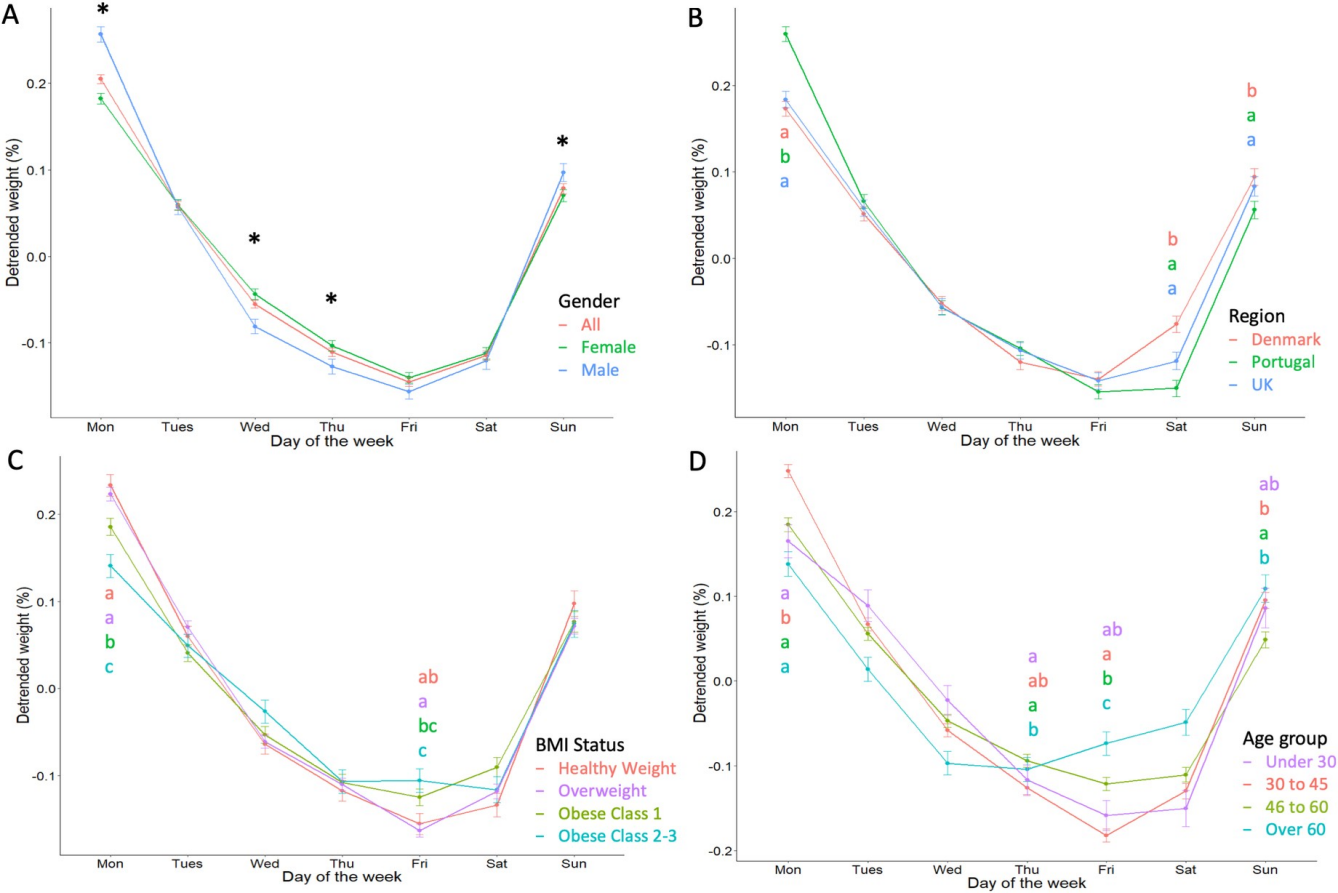
Weekly body weight fluctuations. Weekly body weight fluctuations in all individuals and by gender (A), region (B), BMI status (C) and age group (D). Body weight has been detrended and detrended weight signifies the mean relative deviation from the body weight trend on a given day of the week. Groups are presented by colour, and groups without a letter in common for each given day were significantly different (p<0.05) as tested by multi-factor ANOVA and Tukey’s post hoc. Gender differences (p<0.05) are illustrated using an asterisk.

Within-week patterns were characterized by weekend weight gain and weekday weight reduction in all groups (Fig 4). Means and standard errors are reported in Table 2 with between group comparisons for each day of the week. The results from one-way ANOVA analyses are given in S2 Table. In the whole group, body weight was greatest on Monday, Sunday and Tuesday respectively, and decreased throughout the week with the lowest body weight on Friday. In the whole group, weekly body weight fluctuations of around 0.35% were observed. Both genders displayed similar patterns, though weekly fluctuations were slightly greater in men than women (0.41% vs 0.29%) who had significantly greater weight on Monday and Sunday (p<0.001 for both) and lower weight on Wednesday and Thursday (p<0.01 for both) (Fig 4A).

**Table 2:** Relative weight by day of the week.

Group		Day of the week (relative body weight (%) (se))						
Gender		Mon	Tues	Wed	Thurs	Fri	Sat	Sun
	Men	0.256 (1.07)^a^	0.057 (1.03)	-0.081 (1.02)^a^	-0.127 (1.02)^a^	-0.156 (1.01)	-0.12 (1.07)	0.097 (1.1)^a^
	Women	0.182 (1.12)^b^	0.059 (1.09)	-0.044 (1.09)^b^	-0.104 (1.1)^b^	-0.14 (1.09)	-0.112 (1.12)	0.07 (1.14)^b^
Country	Denmark	0.173 (1.09)^a^	0.051 (1.06)	-0.052 (1.04)	-0.12 (1.06)^a^	-0.14 (1.06)	-0.076 (1.09)^a^	0.094 (1.09)^a^
	Portugal	0.259 (1.05)^b^	0.066 (0.99)	-0.057 (1)	-0.104 (1)^b^	-0.154 (1.01)	-0.15 (1.05)^b^	0.056 (1.1)^b^
	UK	0.184 (1.17)^a^	0.058 (1.16)	-0.056 (1.16)	-0.107 (1.16)^ab^	-0.142 (1.14)	-0.119 (1.17)^b^	0.083 (1.19)^b^
BMI status	Healthy weight	0.233 (1.15)^ab^	0.06 (1.12)	-0.064 (1.11)	-0.118 (1.12)	-0.156 (1.13)^ab^	-0.134 (1.16)	0.098 (1.19)
	Overweight	0.186 (1.04)^a^	0.041 (1.02)	-0.053 (1.02)	-0.108 (1.02)	-0.125 (1)^a^	-0.09 (1.04)	0.077 (1.08)
	Obese C1	0.141 (1.07)^bc^	0.049 (1.08)	-0.027 (1.07)	-0.107 (1.06)	-0.106 (1.06)^bc^	-0.116 (1.08)	0.074 (1.08)
	Obese C2-3	0.223 (1.13)^c^	0.07 (1.08)	-0.061 (1.07)	-0.11 (1.08)	-0.163 (1.08)c	-0.118 (1.12)	0.072 (1.14)
Age group	Under 30 years	0.165 (1.21)^a^	0.089 (1.14)	-0.023 (1.12)	-0.117 (1.11)^ab^	-0.159 (1.11)	-0.15 (1.19)	0.086 (1.25)
	30–45 years	0.248 (1.11)^b^	0.067 (1.08)	-0.058 (1.08)	-0.126 (1.08)^a^	-0.182 (1.09)	-0.13 (1.13)	0.095 (1.15)
	46–60 years	0.138 (1.04)^a^	0.014 (1.04)	-0.097 (1.01)	-0.104 (1.04)^b^	-0.074 (1)	-0.049 (1.03)	0.109 (1.07)
	Over 60 years	0.184 (1.09)^a^	0.056 (1.06)	-0.047 (1.06)	-0.094 (1.06)^ab^	-0.121 (1.06)	-0.111 (1.08)	0.049 (1.1)

Mean (SD) body weight relative to the non-linear trend and standard error following detrending, given for each day of the week. Letters denote results from Tukey’s post-hoc tests which were adjusted for all grouping variables. Only grouping variables which were significant in a type III sum of squares multivariate ANOVA were tested for differences between groups. Letters can be read vertically within a day and group. Groups without a letter in common were significantly different. Full multi-factor ANOVA results are provided in S2 Table.

The weekly pattern was similar for all countries (Fig 4B), though greater weekly fluctuation seemed to be present in Portugal compared to the UK and Denmark (0.41% vs 0.33% vs 0.31%, respectively). The Portugal group had a greater relative weight than both the UK and Denmark groups on Monday (p<0.001 for both) and lower weight than Denmark on Thursday (-0.12 (1.06) % vs -0.1 (1.0) %, p = 0.008). Lastly, Denmark had a greater weight than UK and Portugal on Saturday (p<0.01 for both) and Sunday (p<0.01 for both).

A similar pattern was observed for BMI groups (Fig 4C), though the extent of within-week fluctuation generally decreased with BMI, with the largest fluctuations observed in the healthy weight group followed by the individuals with overweight, individuals with class 1 obesity and lastly individuals with class 2–3 obesity (0.39% vs 0.38% vs 0.31% vs 0.26% respectively). Individuals with class 2–3 obesity showed significantly lower weight on Mondays compared to individuals with overweight, healthy weight (p<0.01 for both) and class 1 obesity (p<0.05). Differences were also observed on Friday where individuals with overweight had significantly lower weight than all individuals with obesity (p<0.001 for both) and individuals with healthy weight had a lower weight than those with class 2–3 obesity (p<0.001).

Differences between age groups were the most detectable (Fig 4D) with the greatest fluctuations coming from 30–45 year old group, followed by under 30s, 46–60 years and lastly over 60 years (0.43% vs 0.32% vs 0.31% vs 0.24% respectively). Individuals aged 30–45 years had a higher weight than all other groups on Monday (p<0.01 for all). On Thursdays, weight was greater in the 46–60 years group compared to 30–45 group (-0.09 (1.06) % vs -0.13 (1.08) %, p = 0.013) and on Fridays weight was greater in those over 46 years than in those aged 30–45 years (p<0.05 for all). On Sunday, greater in those aged 30 to 45 years than in those aged 46 years and above (p<0.05 for both).

### Christmas patterns of body weight

**Fig 5:**
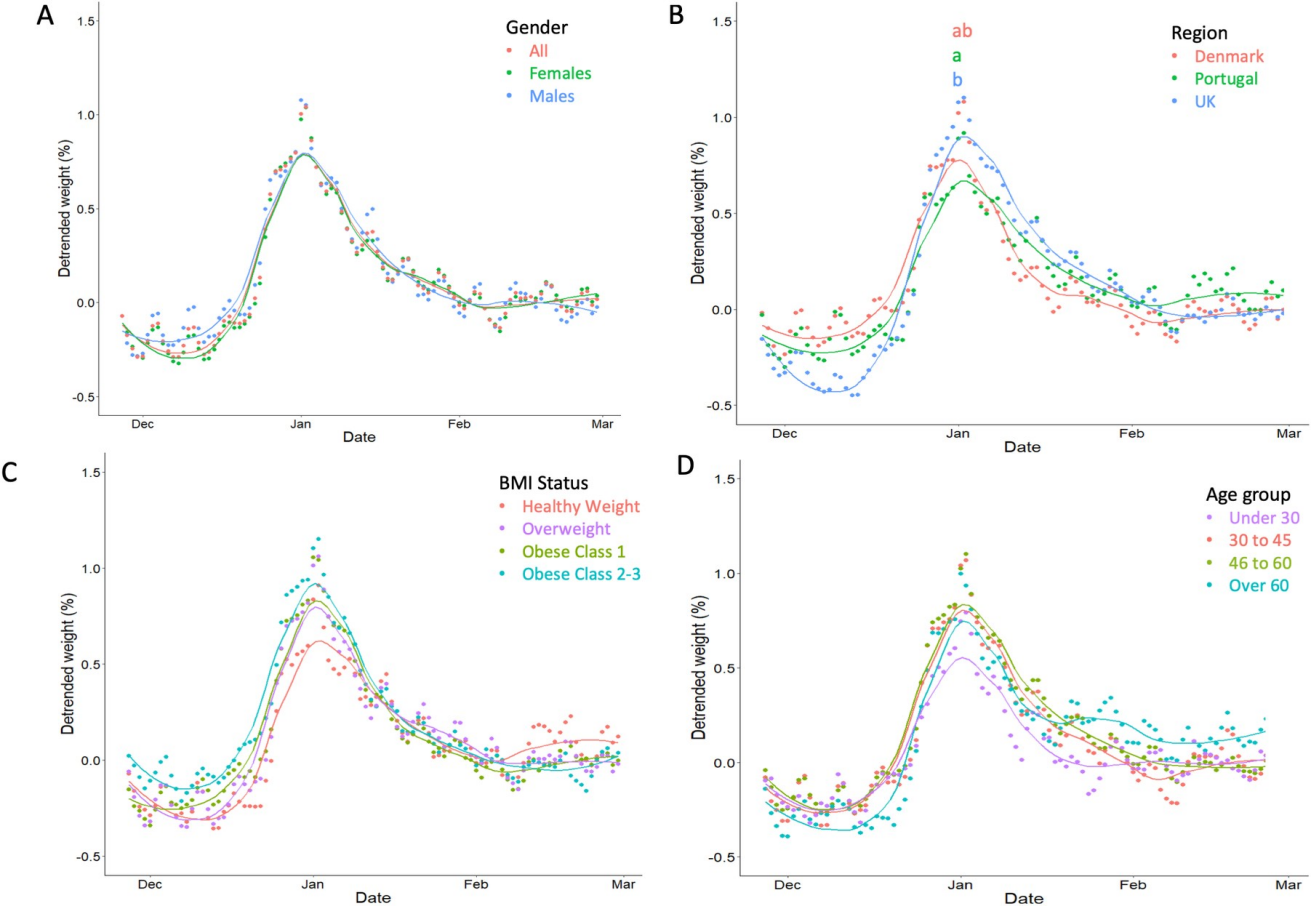
Body weight fluctuation around Christmas. Christmas body weight fluctuations in all individuals and by gender (A), region (B), BMI status (C) and age group (D). Body weight has been detrended and detrended weight signifies the mean relative deviation from the body weight trend on a given day. Groups are presented by colour, and groups without a letter in common for each given day were significantly different as tested by multi-factor ANOVA and Tukey’s post hoc.

Christmas weight gain was observed in all groups (Fig 5). Means and errors are reported in Table 3 with between group comparisons. The results from one-way ANOVA analyses are given in S3 Table. In the whole group, increases of 1.35 (1.74)% body weight were observed, with the lowest weight in the first week of December and the greatest weight on the second day of January. Body weight decreased between January and March though remained at least 0.35% greater than the pre-Christmas weight. Christmas weight gain was similar between men and women (1.30 (1.67)% and 1.37 (1.79)%) (Fig 5A). Between countries, greater body weight gain was observed in the UK compared to the Portugal (1.52 (1.70)% vs 1.13 (1.60)% respectively, p = 0.011), though Denmark was similar to both groups (1.29 (1.65)%, p>0.05 for both comparisons) (Fig 5B). With regards to BMI status (Fig 5C) and age group (Fig 5D), no significant differences in weight gain were observed (p<0.05 for all comparisons).

**Table 3:** Relative holiday weight change by group.

Group	Christmas weight gain (%) (se)
Men	1.30 (1.67)
Women	1.37 (1.79)
Centre	
Denmark	1.29 (1.65) ^ab^
Portugal	1.13 (1.60) ^a^
UK	1.52 (1.70) ^b^
BMI status	
Healthy weight	1.21 (1.78)
Overweight	1.32 (1.85)
Obese C1	1.40 (1.68)
Obese C2-3	1.33 (1.62)
Age group	
under 30 years	1.08 (1.62)
30 to 45 years	1.39 (1.78)
46 to 60 years	1.31 (1.58)
over 60 years	1.40 (1.85)

Mean (SD) body weight relative to the non-linear trend and standard error following detrending around the Christmas period. Letters denote results from Tukey’s post-hoc tests which were adjusted for all grouping variables. Only grouping variables which were significant in a type III sum of squares multivariate ANOVA were probed for differences between groups. Letters can be read vertically within a grouping variable. Groups without a letter in common were significantly different. Full multi-factor ANOVA results are provided in S3 Table.

### Seasonal patterns of body weight

**Fig 6:**
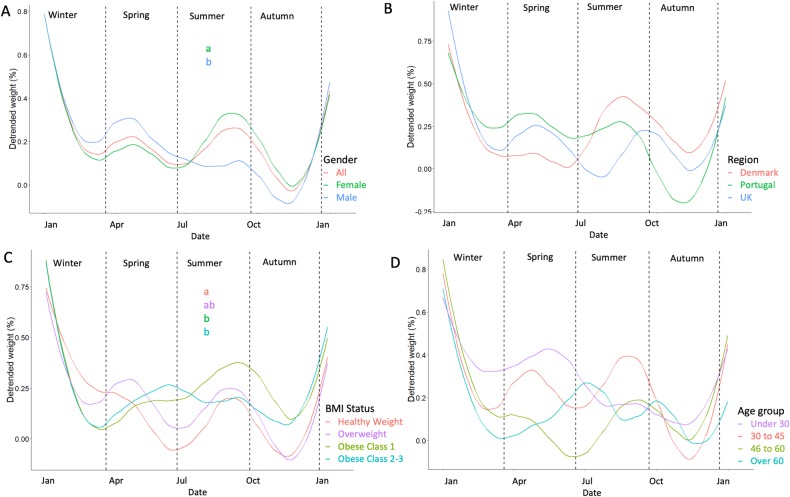
Seasonal body weight fluctuations. Seasonal body weight fluctuations in all individuals and by gender (A), region (B), BMI status (C) and age group (D). Body weight has been detrended and detrended weight signifies the mean relative deviation from the body weight trend on a given day of the year which has been given as a line for each group. Groups are presented by colour, and groups without a letter in common for each given day were significantly different as tested by multi-factor ANOVA and Tukey’s post hoc.

Seasonal patterns in relative body weight are shown in Fig 6 and means and standard errors for relative weight are reported in Table 4 with between group comparisons. The results from one-way ANOVA analyses are given in S4 Table. Following detrending, body weight fluctuated by around 0.8% per year in the whole group, and patterns were largely characterized by Christmas weight gain and loss during the year. Gender differences were observed (Fig 6A); men lost weight and therefore had significantly lower weights during summer, compared to women who gained weight (0.23 (1.32) % vs 0.40 (1.19) %, p = 0.034). Between countries (Fig 6B), no significant differences were observed. During summer, weight was greater in both obese groups (Fig 6C), in comparison to healthy weight individuals (p<0.05 for both). Between age groups, no differences were observed for all seasons (Fig 6D).

**Table 4:** Relative seasonal weight patterns.

		Spring	Summer	Autumn	Winter
Gender	Men	0.28 (1.35)	0.23 (1.32) ^a^	0.02 (1.27)	0.38 (1.14)
	Women	0.21 (1.16)	0.40 (1.19) ^b^	0.13 (1.03)	0.34 (0.91)
Country	Denmark	0.08 (1.42)	0.34 (1.34)	0.21 (1.25)	0.35 (1.09)
	Portugal	0.27 (1.38)	0.32 (1.39)	-0.08 (1.29)	0.32 (1.15)
	UK	0.32 (1.41)	0.39 (1.49)	0.12 (1.22)	0.39 (1.17)
BMI Status	Healthy Weight	0.18 (1.38)	0.06 (1.45) ^a^	-0.02 (1.36)	0.38 (1.35)
	Overweight	0.20 (1.12)	0.31 (1.31) ^ab^	0.04 (1.15)	0.32 (1.01)
	Obese C1	0.27 (1.6)	0.55 (1.70) ^b^	0.24 (1.52)	0.39 (2.05)
	Obese C2-3	0.39 (1.98)	0.55 (2.17) ^b^	0.14 (1.88)	0.37 (3.59)
Age group	Under 30 years	0.43 (2.15)	0.34 (1.82)	0.14 (1.91)	0.41 (1.79)
	30–45 years	0.28 (1.51)	0.46 (1.41)	0.09 (1.21)	0.34 (1.05)
	46–60 years	0.11 (1.39)	0.20 (1.39)	0.09 (1.22)	0.40 (1.01)
	Over 60 years	0.23 (2.00)	0.47 (2.11)	0.08 (1.71)	0.20 (1.78)

Mean (SD) body weight relative to the linear trend and standard error following detrending across different seasons of the year. Multiple years were aggregated. Letters denote results from Tukey’s post hoc tests which were run adjusted for all grouping variables. Only grouping variables which were significant in a type III sum of squares multivariable were probed for differences between groups. Letters can be read vertically within a grouping variable. Groups without a letter in common were significantly different. Full multi-factor ANOVA results are provided in S4 Table.

## Discussion

In the present study we observed weekly fluctuations in the region of 0.35% body weight which were relatively consistent across groups; substantial Christmas weight gain in the region of 1.3% which was not fully compensated for in following months and seasonal patterns which varied between groups and were largely characterized by weight gain during the Christmas and New Year period.

We observed greater body weight around the weekend which was greatest on Monday and decreased throughout the week reaching the lowest weight on Friday, with fluctuations being equal to around 0.35% (around 0.3kg in the present group). Our observations are in line with results from previous research which has shown around 0.17kg fluctuation between Monday and Friday in 48 adults involved in a weight loss intervention in which participants were randomized into either caloric restriction or exercise arms [12]. Similarly, Monday and Friday were identified as the maximum and minimum weight days in an analysis of 80 adults from discrete 4 studies [11]. In the present study we support these observations using a large and diverse population and show replicability in different genders, regions, ages and BMI groups.

Indeed, human behaviour is subject to both biological and environmental rhythms. The 7-day rhythm is consistent and therefore likely associated with predictable changes in behaviour which include (in some samples) weekend reductions in workplace activity [39] and increases in dietary energy density [18] characterised by increased energy, fat and alcohol intake [19] including preferences for sugar sweetened beverages, discretionary/processed foods and fast foods [17]. While it might be expected that different groups may have discrete behavioural responses to the weekly cycle, we observed relatively consistent patterns of weight fluctuation. Two notable exceptions from the overall pattern were evident. First, those over 60 years old tended to show a less prominent weekly cycle. It could be postulated that many individuals over the age of 60 are in retirement and therefore may not show behavioural responses to the weekly cycle. Moreover, as appetite declines in elderly individuals, episodes of excessive intake (which are often around weekends) may become less frequent [41]. Individuals in Portugal tended to maintain their weekday weight reduction from Friday to Saturday, whereas weight gain was observed in the UK and more so Denmark from Friday till Monday. This suggests behavioural changes occur later in the week in Portugal compared to the two other countries and may be reflective of cultural differences.

Weight variability has previously been associated with weight gain and obesity [7,8] potentially due to dysregulated/inconsistent energy balance behaviours and therefore associations between BMI and weekly weight fluctuations may be expected. However, we observed an inverse association between BMI and weekly fluctuation, with healthy weight individuals displaying the greatest weekly fluctuation (0.4% vs 0.27% in individuals with class 2–3 obesity). This observation was made previously by Orsama et al. (2014) and may be explained by the removal of the weight trend [11], meaning that greater weekly fluctuation is reflective of greater weekday compensation for weekend weight gain, whereas lack of compensation results in an upward trend (which is presently removed). This is demonstrated by the fact individuals with obesity had greater weights on Friday.

We observed patterns of weight gain in the region of 1.35% in the whole group (around 1.10kg in the present group) beginning in early December and continuing until the first few days of January. These findings are in line with previous observations reporting around 0.2-1kg weight gain over Christmas in the general population [13]. However, less weight gain (or even weight loss) may occur in individuals engaged in a weight loss or maintenance intervention [13,42]. Therefore, in the present group we observed slightly large Christmas weight gain. One explanation for this observation may be that individuals joining a weight loss maintenance intervention may potentially do so as they are more susceptible to weight gain (i.e. susceptibility to weight gain precedes a weight control attempt [7]), and therefore are more likely to gain weight over the Christmas period.

To quantify Christmas weight gain, previous trials have often relied on a single or a very small number of body weight measurements before and after the Christmas period [25–27,43,44]. This may result in single-measurement error due to normal fluctuations related to total body water, glycogen and other factors. To overcome this, a smoothed time-series of body weight measures over the entire Christmas period and following period was generated, including a minimum of 4 weight measurements in the month before and after Christmas. Interestingly, we observed a partial but incomplete reduction in weight following the new year until March, which remained around 0.35% (or under 0.30kg) greater than before Christmas. This evidence supports the hypothesis that holiday weight gain may be a factor contributing to long-term weight gain [45].

Holiday weight gain was greater in individuals from the UK than in those from Portugal. In a previous study of holiday weight gain across countries in 2,924 adults, authors reported greater weight gain in individuals based in Germany (0.6%) compared to the United States (0.4%) and Japan (0.5%) [46] following detrending of the body weight data; and Christmas weight gain as low as 0.2% has been observed in Spain [47]. Together, these results infer that cultural differences in the behavioural response to the holiday period are present.

No differences were observed between BMI groups. This is inconsistent with previous literature suggesting that more weight is gained by individuals with overweight and obesity in comparison to those with normal weight [13,26], as well as the hypothesis that holiday weight gain contributes to obesity [45]. However, we used relative rather than absolute weight and this accounts for differences in initial body size and may have exaggerated weight gain in heavier individuals in previous studies. Further explanation comes from the fact that the energy cost of weight gain is greater in heavier individuals, due to differences in proportions of fat and fat-free mass gained, and differences in the energy content of both tissues [48]. Furthermore, it is likely that individuals with obesity are more likely to be gaining weight at any given period than those with normal weight and to correct for this we removed the overall trend in body weight over a longer period in order to determine the response to the Christmas period. Lastly, weight gain was similar between all individuals irrespective of age, though generally younger individuals seemed to gain less weight during the period. Interestingly, all groups remained at elevated body weights up to 2 months into the new year, suggesting that weight gained at Christmas is not fully compensated for in the subsequent period. Together, these results can inform potential targets for future weight control interventions, such as self-monitoring intervention around Christmas [49].

Seasonal patterns were less consistent and the most obvious pattern of weight gain in December and January is likely to be an outcome of the Christmas effect. It is worth noting that observed errors in group means were large, suggesting that these seasonal patterns are irregular, inconsistent and not defined by the grouping variables used presently. Previous studies have reported seasonal patterns in body weight, with one study reporting fluctuations of around 0.5kg throughout the year with a peak in winter and trough in summer in a sample of 593 American individuals [28]; another study reported a 1.2% increase in weight between fall and winter followed by a 0.6% decrease from winter to spring in 248 American individuals engaged in a weight loss intervention which promoted daily self-weighing [14]. Again, it is worth noting these studies did not adjust for weight gain or loss throughout the year which may confound seasonal fluctuations. In a comprehensive analysis involving yearly detrending and aggregation of data from 10,000 randomly selected digital smart scale users from 7 countries around the world [15], authors reported seasonal fluctuations in the region of 0.3% body weight which were inconsistent between region though every country displayed a clear holiday effect (similar to the present results).

Gender differences were observed in summer, characterized by a reduction in body weight during the season in men and an increase in women. It is possible that this is due to gender differences in physical activity, whereby men are more predisposed to partake in physical activity [50] and physical activity increases in summer [51]. Together, these may influence a negative energy balance in men but not women during summer. Further differences were observed between BMI groups in summer; healthy weight individuals had lower relative weight than those in obese groups. Again this could be explained by changes in physical activity during the summer period, as those lower in BMI generally have greater levels of physical activity [52]. No differences between countries were observed, though individuals in the UK showed a reduction in weight going from spring to summer, whereas individuals in Denmark (and less so Portugal) gained weight during this period. Further research on examining seasonal fluctuations in energy balance behaviors may help us understand some of these differences better.

The present study has several benefits, including consistent measurement of bodyweight for up to two years, which allowed for employment of time series modelling which would be inappropriate where weight data was infrequent. Further, we had a large sample size which ranged between 1,062 participants (for describing Christmas patterns) to 1,421 (for describing within-week patterns). This allowed us to explore group differences in fluctuation patterns, which, to our knowledge, have not previously been examined. Next, individuals weighed themselves on average around 2.5 times per week over 566–607 days, and restrictions were put in place to exclude participants with excessive missing data. Lastly, we adjusted our multivariate ANOVA models for each grouping variable when testing for differences between groups to control for potential lack of balance.

There were also limitations to the current analysis. All individuals were engaged in a weight loss maintenance intervention and therefore our observations may not be representative of the general population. Adherence to self-monitoring has previously been associated with reduced weight fluctuation [53] and therefore patterns may be more pronounced in individuals not regularly self-weighing. However, individuals in the present group are more likely to struggle with regulating body weight and therefore may show more pronounced patterns of fluctuation. Recruitment to the intervention was rolling, therefore initiation of self-monitoring began at different stages of the year in different individuals and may have influenced weighing or energy balance behaviours. Next, we grouped individuals by baseline variables on which data is easy to collect, but it may be that these characteristics are not necessarily related to weight fluctuation and as such, further exploration of psychological and behavioural variables is advised. Continuous tracking of physical activity may facilitate improved understanding of fluctuations in body weight, though energy expenditure estimates from these devices may lack precision [54,55]. We were unable to tell whether individuals adhered to the self-weighing guidance provided (i.e. first thing in the morning in light or no clothing and an empty bladder). However, it is unlikely that lack of adherence to this guidance would produce the body weight patterns observed. Next, while we observed relatively high adherence to self-weighing, missing data was present and imputation using an exponentially weighted moving average was conducted (informed by a results of an unpublished simulation study on body weight data imputation by this group) in the case of Christmas and seasonal analyses, which is second to using true data. Lastly, we had less than three years of data between 2017 and 2019 and therefore seasonal patterns had limited replicability, and we were not able to investigate year-to-year differences in seasonal patterns.

Using frequent measurements of body weight collected by electronic smart scales, we applied time-series modelling in a large and diverse sample of individuals engaged with a weight loss maintenance intervention to show clear patterns of weekend weight gain and weekday weight loss; Christmas weight gain (which was not fully compensated for in following months) and minor and inconsistent seasonal patterns. Weekly patterns differed slightly between groups though consistent weekend effects were observed for all. Christmas weight gain was more pronounced in the UK than Portugal and differences in seasonal patterns were minor in magnitude. The present study highlights the influence of the temporal environment on energy balance behaviors, and how these may interact with individual characteristics and cultural differences. These results may inform future interventions aimed at reducing periods of overconsumption and weight gain, particularly in specific groups. Future research employing smart scales should consider the impact of body weight fluctuations on weight outcomes.

## Supporting information

S1 Fig Imputation by exponentially weighted moving average. (DOCX)

S2 Fig Detrending process using non-linear and linear trends. (DOCX)

S1 Table Outlier removal criteria. (DOCX)

S2 Table ANOVA results for day of week analysis between groups. (DOCX)

S3 Table ANOVA results for Christmas weight gain between groups. (DOCX)

S4 Table ANOVA results seasons analysis between groups. (DOCX)
