# Supplementary material for: Weekly, seasonal and holiday body weight fluctuation patterns among individuals engaged in a European multi-centre behavioural weight loss maintenance intervention
Source: PLoS One. 2020 Apr 30;15(4):e0232152. doi: 10.1371/journal.pone.0232152 (PMC7192384; doi:10.1371/journal.pone.0232152)
Supplement: S1 Fig — (DOCX) [file pone.0232152.s001.docx]

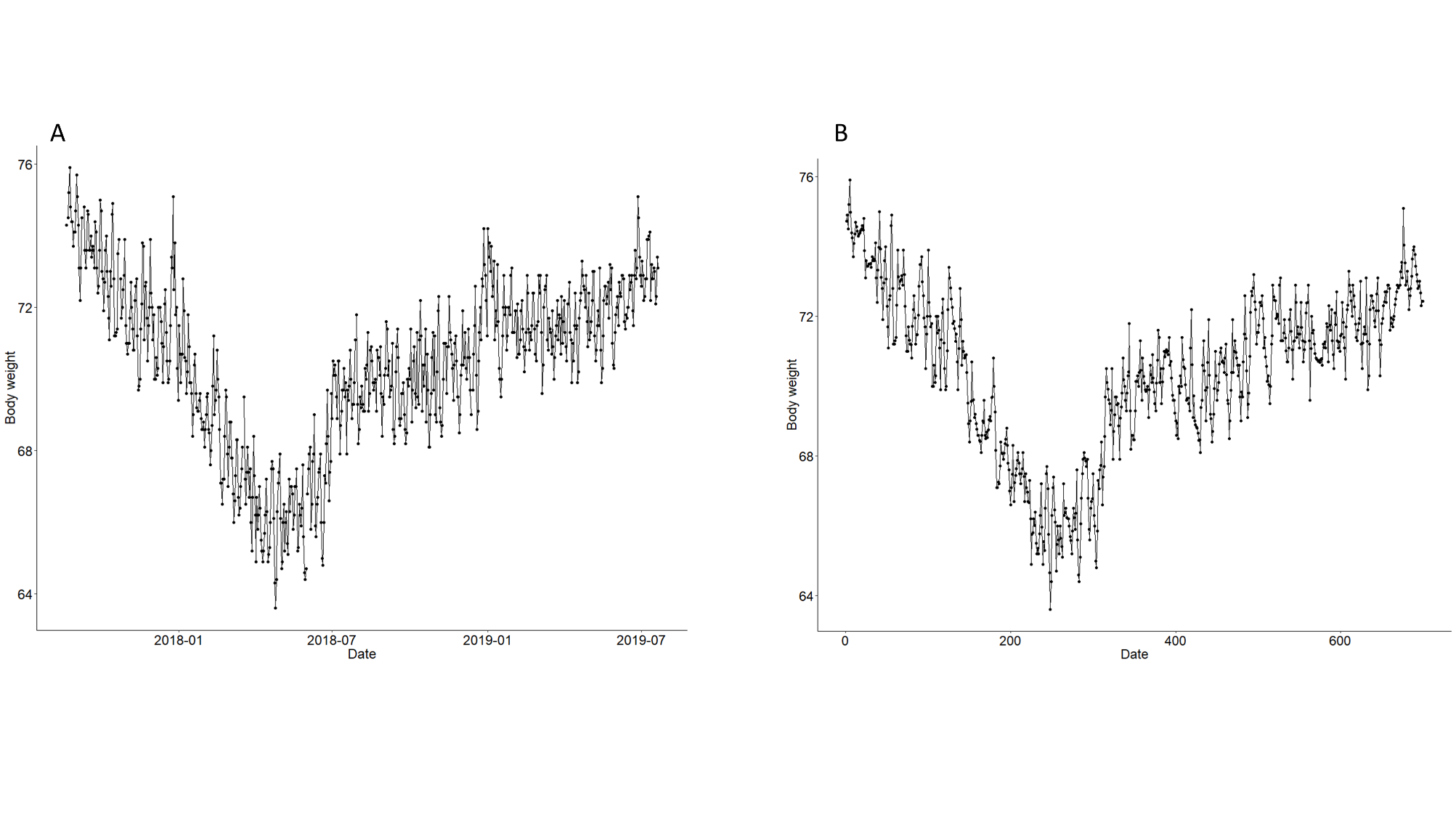


Supplementary figure 1: Imputation by exponentially moving average. In figure (A), true data is presented from a single participant which illustrates body weight fluctuating on a weekly basis. In figure (B), we removed 50% of the true data using a missing completely at random (MCAR) strategy and imputed the missing data using an exponentially weighted moving average with a 7 day window. From this, we can see some of the day-to-day variability is reduced in figure (B) and therefore we used only true data for the within-week analysis. However, larger/longer duration fluctuations are still present and therefore imputation was used in seasonal and holiday analyses.
