# Supplementary material for: Weekly, seasonal and holiday body weight fluctuation patterns among individuals engaged in a European multi-centre behavioural weight loss maintenance intervention
Source: PLoS One. 2020 Apr 30;15(4):e0232152. doi: 10.1371/journal.pone.0232152 (PMC7192384; doi:10.1371/journal.pone.0232152)
Supplement: S2 Fig — (DOCX) [file pone.0232152.s002.docx]

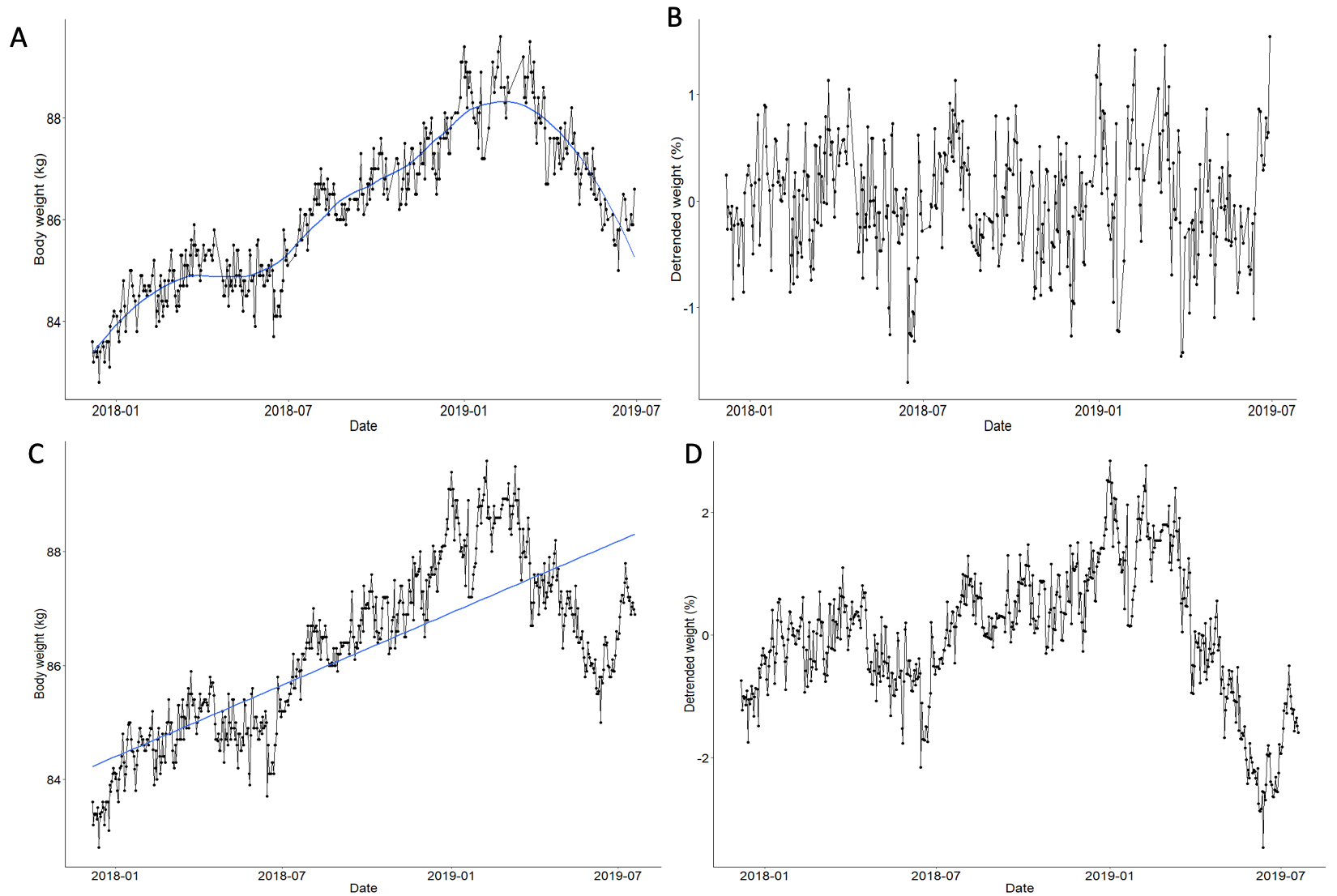


Supplementary figure 2. Detrending process using non-linear and linear trends. A single participants body weight data is presented to illustrate the process of non-linear and linear detrending. In figure (A) a non-linear loess regression smoother is fitted to the data with a span of 0.5. In figure (B), the non-linear trend is subtracted from the weight values, and relative deviations (%) from the trend are produced for each timepoint. In figure (C) a linear regression is fitted to the data and in figure (B), the linear trend is subtracted from the weight values, and relative deviations (%) from the trend are produced for each timepoint.
