# Supplementary material for: Weekly, seasonal and holiday body weight fluctuation patterns among individuals engaged in a European multi-centre behavioural weight loss maintenance intervention
Source: PLoS One. 2020 Apr 30;15(4):e0232152. doi: 10.1371/journal.pone.0232152 (PMC7192384; doi:10.1371/journal.pone.0232152)
Supplement: S1 Table — (DOCX) [file pone.0232152.s003.docx]

Supplementary table 1. Maximum limits of weight change allowed, outwith which data are removed as outliers. Limits are informed by physiologically plausible observations under conditions of substantial under or overfeeding, as referred to in-text.

| **Supplementary table 1.** Limits of weight change within a given time period | |
| --- | --- |
| Change in body weight | Duration |
| ± 5% | 1 week |
| ± 10% | 4 weeks |
| ± 15% | 8 weeks |
| ± 20% | 12 weeks |
