# Supplementary material for: Weekly, seasonal and holiday body weight fluctuation patterns among individuals engaged in a European multi-centre behavioural weight loss maintenance intervention
Source: PLoS One. 2020 Apr 30;15(4):e0232152. doi: 10.1371/journal.pone.0232152 (PMC7192384; doi:10.1371/journal.pone.0232152)
Supplement: S2 Table — (DOCX) [file pone.0232152.s004.docx]

| Supplementary table 2. ANOVA results of group differences in detrended body weight between groups for days of the week | | | | |
| --- | --- | --- | --- | --- |
| Day | Group | Sum of Squares | F value | P value |
| Mon | Gender | 16.629 | 13.661 | <0.001 |
|  | Centre | 12.425 | 5.104 | 0.006 |
|  | BMI Status | 29.348 | 8.036 | <0.001 |
|  | Age Group | 44.288 | 12.127 | <0.001 |
|  |  |  |  |  |
| Tues | Gender | 0.855 | 0.742 | 0.389 |
|  | Centre | 1.066 | 0.463 | 0.630 |
|  | BMI Status | 6.423 | 1.859 | 0.134 |
|  | Age Group | 3.336 | 0.965 | 0.408 |
|  |  |  |  |  |
| Wed | Gender | 16.588 | 14.591 | <0.001 |
|  | Centre | 2.764 | 1.216 | 0.296 |
|  | BMI Status | 7.761 | 2.276 | 0.078 |
|  | Age Group | 4.33 | 1.269 | 0.283 |
|  |  |  |  |  |
| Thurs | Gender | 8.852 | 7.68 | 0.006 |
|  | Centre | 10.292 | 4.465 | 0.012 |
|  | BMI Status | 1.826 | 0.528 | 0.663 |
|  | Age Group | 10.369 | 2.998 | 0.029 |
|  |  |  |  |  |
| Fri | Gender | 0.469 | 0.411 | 0.521 |
|  | Centre | 1.362 | 0.597 | 0.55 |
|  | BMI Status | 20.789 | 6.077 | <0.001 |
|  | Age Group | 46.719 | 13.656 | <0.001 |
|  |  |  |  |  |
| Sat | Gender | 1.879 | 1.543 | 0.214 |
|  | Centre | 29.812 | 12.241 | <0.001 |
|  | BMI Status | 4.029 | 1.103 | 0.346 |
|  | Age Group | 7.798 | 2.135 | 0.094 |
|  |  |  |  |  |
| Sun | Gender | 15.447 | 12.165 | <0.001 |
|  | Centre | 27.909 | 10.99 | <0.001 |
|  | BMI Status | 8.214 | 2.156 | 0.091 |
|  | Age Group | 22.717 | 5.963 | <0.001 |

**Supplementary table 2**. Results from multivariate ANOVA with type III sum of squares showing differences in mean body weight relative to the non-linear trend between groups for each day of the week
