# Supplementary material for: Weekly, seasonal and holiday body weight fluctuation patterns among individuals engaged in a European multi-centre behavioural weight loss maintenance intervention
Source: PLoS One. 2020 Apr 30;15(4):e0232152. doi: 10.1371/journal.pone.0232152 (PMC7192384; doi:10.1371/journal.pone.0232152)
Supplement: S3 Table — (DOCX) [file pone.0232152.s005.docx]

| Supplementary table 3. ANOVA results of group differences in detrended body weight gain during the Christmas period | | | | |
| --- | --- | --- | --- | --- |
| Predictor variable | Model variables | Sum Sq | F value | Pr(>F) |
|  |  |  |  |  |
| Gender | Gender | 0.003 | 0.001 | 0.974 |
|  | Centre | 22.082 | 3.603 | 0.028 |
|  | BMI Status | 17.752 | 1.931 | 0.123 |
|  | Age Group | 13.091 | 1.424 | 0.234 |
|  |  |  |  |  |
| Centre | Gender | 0.551 | 0.201 | 0.654 |
|  | Centre | 24.768 | 4.53 | 0.011 |
|  | BMI Status | 7.277 | 0.887 | 0.447 |
|  | Age Group | 11.024 | 1.344 | 0.259 |
|  |  |  |  |  |
| BMI Status | Gender | 1.413 | 0.454 | 0.501 |
|  | Centre | 7.843 | 1.258 | 0.285 |
|  | BMI Status | 3.843 | 0.411 | 0.745 |
|  | Age Group | 10.833 | 1.159 | 0.324 |
|  |  |  |  |  |
| Age Group | Gender | 0.178 | 0.062 | 0.803 |
|  | Centre | 25.051 | 4.359 | 0.013 |
|  | BMI Status | 5.38 | 0.624 | 0.6 |
|  | Age Group | 9.373 | 1.087 | 0.354 |

**Supplementary table 3**. Results from multivariate ANOVA with type III sum of squares showing differences in mean body weight gain relative to the non-linear trend between groups.
