# Supplementary material for: Weekly, seasonal and holiday body weight fluctuation patterns among individuals engaged in a European multi-centre behavioural weight loss maintenance intervention
Source: PLoS One. 2020 Apr 30;15(4):e0232152. doi: 10.1371/journal.pone.0232152 (PMC7192384; doi:10.1371/journal.pone.0232152)
Supplement: S4 Table — (DOCX) [file pone.0232152.s006.docx]

| Supplementary table 4. ANOVA results of group differences in detrended body weight between groups for days of the week | | | | |
| --- | --- | --- | --- | --- |
| Season | Group | Sum of squares | F value | P value |
| Spring | Gender | 0.286 | 0.058 | 0.81 |
|  | Centre | 25.333 | 2.565 | 0.077 |
|  | BMI Status | 10.441 | 0.705 | 0.549 |
|  | Age Group | 21.391 | 1.444 | 0.228 |
|  |  |  |  |  |
| Summer | Gender | 27.957 | 4.493 | 0.034 |
|  | Centre | 6.422 | 0.516 | 0.597 |
|  | BMI Status | 88.657 | 4.749 | 0.003 |
|  | Age Group | 44.246 | 2.37 | 0.069 |
|  |  |  |  |  |
| Autumn | Gender | 0.523 | 0.135 | 0.713 |
|  | Centre | 21.186 | 2.738 | 0.065 |
|  | BMI Status | 13.489 | 1.162 | 0.323 |
|  | Age Group | 4.581 | 0.395 | 0.757 |
|  |  |  |  |  |
| Winter | Gender | 2.29 | 0.58 | 0.446 |
|  | Centre | 5.693 | 0.721 | 0.486 |
|  | BMI Status | 3.918 | 0.331 | 0.803 |
|  | Age Group | 14.256 | 1.204 | 0.307 |

**Supplementary table 4**. Results from multivariate ANOVA with type III sum of squares showing differences in mean body weight relative to the non-linear trend between groups for each day of the week
